# The Association Between Premature Ejaculation and Non-Dipper Blood Pressure: A Cross-Sectional Study

**DOI:** 10.3390/jcm14238408

**Published:** 2025-11-27

**Authors:** Yaşar Turan, Abdullah Gürel, Elif Turan, Mustafa Yolcu, Güney Erdoğan, Ahmet Karagöz, Mehmet Caniklioğlu

**Affiliations:** 1 Department of Cardiology, Faculty of Medicine, Samsun University, Samsun 55100, Turkey; 2 Department of Urology, Faculty of Medicine, Afyonkarahisar Health Sciences University, Afyonkarahisar 03200, Turkey; abdullahgurel@hotmail.com; 3 Department of Endocrinology, Faculty of Medicine, Samsun University, Samsun 55100, Turkey; 4 Department of Cardiology, Faculty of Medicine, İstanbul Yeni Yüzyıl University, İstanbul 34245, Turkey; 5 Department of Cardiology, Samsun Education and Research Hospital, Samsun 55100, Turkey; 6 Department of Urology, Faculty of Medicine, Yozgat Bozok University, Yozgat 66200, Turkey

**Keywords:** premature ejaculation, sexual dysfunction, blood pressure variability, autonomic nervous system

## Abstract

**Background/Objectives**: Premature ejaculation (PE) is one of the most common sexual problems in men. Autonomic nervous system (ANS), which is an important determinant of circadian changes in blood pressure (BP), also has a mechanism that controls ejaculation. We aimed to investigate the relationship between PE and BP variability. **Methods**: This cross-sectional study included 80 normotensive patients with PE and 80 healthy volunteers. All the participants underwent 24-h ambulatory BP measurement. Participants were categorized into two groups based on the percentage of nocturnal BP dipping: the dipper BP (DBP), and non-dipper BP (NDBP) groups. **Results**: The frequency of the NDBP pattern was significantly higher in the PE group compared to the control group (48% vs. 28%, *p* = 0.009). In the multivariate logistic regression analysis, the NDBP pattern remained significantly associated with PE [odds ratio: 0.399, 95% confidence interval: (0.207–0.770), *p* = 0.006]. Within the PE group premature ejaculation diagnostic tool (PEDT) scores were significantly higher in individuals with NDBP than individuals with DBP (15.62 ± 2.85 vs. 14.32 ± 2.65, *p* = 0.038). **Conclusions**: The frequency of the NDBP pattern was significantly higher in the PE group among normotensive individuals. Additionally, within the PE group, PEDT scores were significantly higher in individuals with the NDBP pattern. A multidisciplinary approach and large-scale prospective studies are necessary to fully elucidate the relationship between PE and the cardiovascular system.

## 1. Introduction

Premature ejaculation (PE) is one of the most common sexual problems in men [1]. PE is defined as the occurrence of ejaculation earlier than the desired time during sexual activity [2]. Clinically, PE is defined as involuntary ejaculation that often or always occurs before or within 1 min of vaginal penetration [3]. Because the definition is not very objective, it is estimated that 20% to 30% of men are affected by PE [4,5].

Semen secretion is innervated by the thoracolumbar (T10–L2) adrenergic sympathetic nerves. Ejaculation is a complex reflex mediated by the influence of the peripheral and central nervous systems [6]. The lumbar (L3–L4) segment of the spinal cord is responsible for ejaculation and contains lumbar spinothalamic (LST) cells. Activation of LST cells causes ejaculation. LST cells coordinate parasympathetic and sympathetic stimulation during ejaculation [7]. Supraspinal centers control the excitatory and inhibitory messages that act on the spinal reflex arcs [8], and conditions affecting the parasympathetic and sympathetic systems that influence ejaculation can cause PE [9].

The human body has a unique diurnal rhythm and exhibits different metabolic activities to maintain day-night synchrony. These activities result in changes in metabolism, sleep–wake cycles, body temperature, and blood pressure (BP) throughout the day. With regular night-time sleep, BP is expected to decrease during the night while resting, rise sharply in the morning, and reach a peak in the late afternoon [10]. The autonomic nervous system (ANS), particularly the sympathetic nervous system (SNS), is the most important determinant of circadian changes in BP. However, other neurohormonal systems that regulate BP also influence circadian rhythms and may contribute to diurnal BP variability. During sleep, SNS dominance decreases, and parasympathetic nervous system (PNS) activation increases, resulting in a BP-lowering effect [11]. Under normal conditions, night-time BP is expected to be 10% lower than daytime BP. A decrease in night-time BP of more than 10% is defined as dipper BP (DBP). If this decrease remains below 10%, it is called non-dipper BP (NDBP). Studies have shown that in hypertensive individuals, the risk of cardiovascular disease (CVD) and organ damage is significantly increased in individuals with NDBP compared with those with DBP [12]. There is limited data on whether nocturnal NDBP in normotensive individuals is a significant contributor to potential CVD risk, and this is a current topic of research. 

The relationship between PE and ANS activity remains a current research topic. A few multidisciplinary studies have evaluated the relationship between PE and the cardiovascular implications of ANS activity. In this context, a study evaluated the relationship between PE and heart rate variability, an indicator of the balance between the SNS and PNS, and reported increased markers of ANS dysfunction in the PE group [13]. Furthermore, post-exercise heart rate recovery is reported to be impaired in patients with PE [14]. Another study reported higher orthostatic intolerance symptoms in PE patients [15]. Although all these parameters are important for determining the cardiovascular implications of ANS dysfunction, there is no data examining the relationship between BP variability and PE, in which ANS function plays a vital role. Given the limited data on this topic, our primary hypothesis was that NDBP pattern would be more common among patients with PE than in the control group. Our secondary hypothesis was that PE severity scores would be higher among individuals with NDBP in the PE group.

In this study, we aimed to investigate the relationship between NDBP and PE in normotensive individuals.

## 2. Materials and Methods

This study had a cross-sectional design. An a priori power analysis indicated that a minimum of 55 participants per group would be required to achieve 90% statistical power at an alpha level of 0.05. The current study included 80 PE patients with office BP measurements below 140/90 mmHg, who were not taking any antihypertensive medication and who presented to the Urology outpatient clinic of Bozok University Faculty of Medicine. A total of 80 healthy participants without PE or hypertension, recruited through voluntary enrollment and routine health examinations, were included in the control group. The study was conducted in accordance with the Declaration of Helsinki. The Yozgat Bozok University Clinical Research Ethics Committee approved the study (2017-KAEK-189_2019.06.19_06). Written informed consent was obtained from all participants before participating in the study. Considering that both hypertension and antihypertensive treatment could affect the study parameters, the study was designed with normotensive individuals. Participants in both groups were heterosexual men who had regular sexual intercourse for the past 6 months. Participants with hormonal disorders, erectile dysfunction (International Index of Erectile Function-5 < 22) [16], prostatitis, Peyronie’s disease, hypospadias, known CVD, diabetes, hematological disease, psychiatric disease, liver or kidney failure, and those receiving PE treatment were excluded. Of the patients initially included in the study, 32 patients diagnosed with hypertension on 24-h ambulatory BP measurement (ABPM), 16 patients with inadequate ABPM records, and 18 patients with missing data were excluded. A total of 160 participants were included in the final analysis.

The diagnosis of PE was made based on detailed clinical history and self-reported symptoms. In addition, each patient was asked to measure intravaginal ejaculatory latency time (IELT) using a stopwatch [17]. The premature ejaculation diagnostic tool (PEDT) was administered to all participants [18]. Patients with IELTs of less than 1 min were included in the PE group. The control group consisted of healthy volunteers with no PE complaints, with a concomitant PEDT score ≤ 8 [19,20]. Demographic characteristics of all participants were recorded. Body mass index (BMI) was calculated using the formula (weight in kg)/(height in meters)^2^. After a 12-h fast, all participants underwent blood collection to determine fasting blood sugar, lipid profile, liver and kidney function, and uric acid levels, as well as conduct a hemogram.

### 2.1. Blood Pressure Measurement

Office BP was defined as the average of two sphygmomanometer readings taken in a sitting position at least 5 min apart. ABPM was recorded at 30-min intervals over 24 h using an oscillometric cuff (Mobil-O-Graph, I.E.M. GmbH, Stolberg, Germany) on the patient’s non-dominant arm. None of the patients took additional medications that could affect circadian BP. Sleep and wake times were calculated based on patient data. The formula 100 × [1 − (sleep systolic BP/awake systolic BP)] was used to calculate the nocturnal BP decrease (%). The DBP pattern was defined as a decrease in systolic and diastolic BP of more than 10%. Decreases of less than 10% were defined as NDBP [21]. The ABPM records of all participants were evaluated by the same cardiologist, who was blinded to the clinical data.

### 2.2. Statistical Analysis

Statistical analyses were performed using SPSS Statistics, version 27.0 (SPSS Inc., Chicago, IL, USA). The distribution of continuous variables was assessed using the Kolmogorov–Smirnov test. Comparative analyses were conducted between the PE group and the controls, using Student’s t-test for normally distributed continuous variables, the Mann-Whitney U-test for non-normally distributed continuous variables, and the chi-square test for categorical data, to assess the primary hypothesis. Additionally, univariate and multivariate logistic regression analyses were performed to identify the factors influencing PE status. Variables with *p*-values < 0.25 in the univariate regression model were incorporated into the multivariate analysis, with PE status as the dependent variable. To test our secondary hypothesis, patients in the PE group were divided into two groups: DBP and NDBP. The difference in PEDT scores between these two groups was analyzed using Student’s t-test. *p* values < 0.05 were considered statistically significant.

## 3. Results

**Figure 1:**
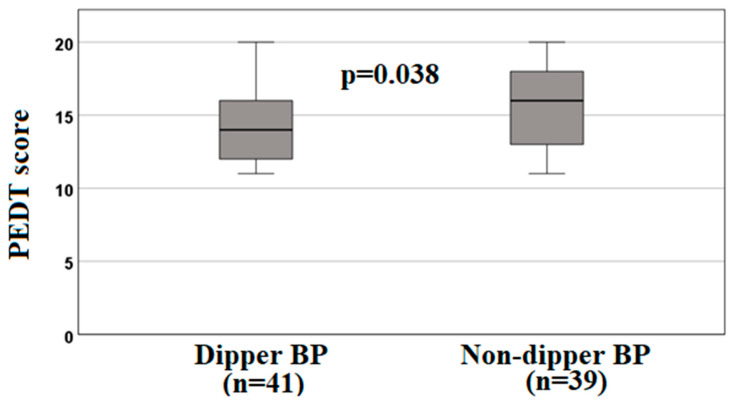
Comparison of premature ejaculation diagnostic tool (PEDT) scores among the individuals with dipper (*n* = 41) and non-dipper (*n* = 39) blood pressure patterns in the premature ejaculation group (*n* = 80). Values are mean ± SD and compared by Student’s t-test. A *p*-value of <0.05 was considered statistically significant.

The two groups were similar in terms of age, smoking status, and BMI (*p* > 0.05). Both groups had a similar 24-h mean systolic BP, 24-h mean diastolic BP, daytime mean systolic BP, daytime mean diastolic BP, and mean systolic and diastolic BP during sleep values (*p* > 0.05). There was no significant difference between the groups in terms of glucose, lipid profile, uric acid, and hemogram parameters (*p* > 0.05). The PEDT scores were significantly higher in the PE group than in the controls (3.50 ± 2.23 vs. 14.95 ± 2.81, *p* < 0.001). The frequency of the NDBP pattern was significantly higher in the PE group compared with the control group (48% vs. 28%, *p* = 0.009) (Table 1). In the multivariate logistic regression analysis, the NDBP pattern remained significantly associated with PE [odds ratio: 0.399, 95% confidence interval: (0.207–0.770), *p* = 0.006] (Table 2). The participants in the PE group were divided into two groups based on their BP patterns: the DBP group and the NDBP group. The PEDT scores were significantly higher in the NDBP group compared with the DBP group (15.62 ± 2.85 vs. 14.32 ± 2.65, *p* = 0.038) (Figure 1).

## 4. Discussion

The study’s main findings were that the frequency of individuals with the NDBP pattern in the PE group was significantly higher than in the control group. Furthermore, within the PE group, PEDT scores were significantly higher in individuals with the NDBP pattern than in individuals with the DBP pattern.

The pathophysiology of PE remains incompletely elucidated. Many psychological and biological factors may contribute to the development of PE [22]. Ejaculation is a complex physiological process influenced by both the autonomic and somatic nervous systems. Ejaculation consists of two phases: emission and expulsion. The organs involved in the emission phase interact intensely with the ANS. The emission phase occurs primarily due to SNS activity [23]. During the second phase of ejaculation, the expulsion phase, seminal fluid reaching the posterior urethra is expelled through the external meatus, triggered by a spinal cord reflex. Although the duration of the emission phase can vary, ejaculation cannot be postponed once the seminal fluid reaches the posterior urethra [24]. In this context, the emission phase is the primary phase affecting PE.

If we assume a relationship between SNS dysfunction and PE, this is likely due to SNS hyperactivity affecting the emission phase. In this context, ANS dysfunction, which has effects throughout the body, may also affect normal ejaculation through similar mechanisms. While the organs involved in the emission phase are in close contact with both sympathetic and parasympathetic nerves, this phase is primarily under the control of the SNS [23]. In the second phase of ejaculation, expulsion, the seminal fluid passes from the posterior urethra to the external urethral meatus via a spinal cord reflex. Although emissions can be controlled to some extent, ejaculation is inevitable once the seminal fluid reaches the posterior urethra [24]. Therefore, we can speculate that the difference in IELT duration between individuals is primarily due to differences in the emission phase. Furthermore, if there is a relationship between ANS dysfunction and PE, it may be due to SNS hyperactivity during the emission phase.

The primary reason for researchers’ interest in non-dipper hypertension is its close association with cardiovascular mortality, morbidity, and end-organ damage [25]. Studies report the adverse cardiovascular effects of NDBP patterns, even in normotensive individuals [26,27,28]. Over the past decade, numerous studies have examined the mechanisms underlying DBP and NDBP patterns [25,29]. Furthermore, the relationship between NDBP pattern and other organ systems is a current research topic. 

The SNS and PNS operate in balance within the body. When this balance is disrupted, the cardiovascular system is among the first to be affected. Increased sympathetic activity and/or decreased parasympathetic activity can be associated with many cardiovascular events, including cardiac arrhythmias, BP changes, and sudden cardiac death [25,30]. Furthermore, studies on hypertension and autonomic functions have shown that patients with an NDBP pattern have an overactive SNS compared with other groups [25]. Another study indicates that a dominant SNS tone, rather than a parasympathetic tone, can cause an NDBP pattern [31].

In our study conducted with normotensive individuals, the NDBP pattern frequency was significantly higher in the PE group. Additionally, within the PE group, PEDT scores were significantly higher in individuals with the NDBP pattern. Several studies exist on the relationship between cardiovascular indicators of ANS function and PE. A study by Özilhan et al. reported higher orthostatic intolerance symptoms in PE patients [15]. Akkoç et al. reported significantly higher P-wave dispersion in individuals with PE [32]. Another study evaluated heart rate variability in PE patients and reported increased indicators of ANS dysfunction and sympathetic overactivity, which may lead to PE [13]. Another study by Turan et al. demonstrated that heart rate recovery after exercise was impaired in the PE group [14].

No data is available evaluating the relationship between PE and BP variability, a parameter closely related to ANS function. In this context, when considered together with previous data, the observed correlation between the NDBP pattern and PE, as well as the higher PEDT scores in the NDBP group among patients with PE, may support the hypothesis that ANS dysfunction is a factor in the development of PE. Furthermore, it may be postulated that ANS dysregulation could underlie both PE and the NDBP pattern through a similar pathophysiological pathway. Specifically, a shift in the balance between sympathetic and parasympathetic tone, which is one of the possible mechanisms of NDBP, toward a sympathetic tone may also lead to PE through a similar mechanism. Considering the effect of sympathetic activity on the emission phase of ejaculation, the already increased sympathetic activity in these individuals provides a biologically plausible mechanism and a possible etiological factor for PE. However, it should not be forgotten that causality cannot be definitively established from the observational relationship obtained in our study.

From a multidisciplinary perspective, it can be argued that the NDBP pattern and PE may represent distinct organ-system findings related to the ANS. When evaluating patients with PE, a broader perspective, including consideration of other organ systems, can be highly beneficial for optimizing current treatment, developing new treatment strategies, and better assessing patients’ long-term cardiovascular risk. Prospective, large-scale studies on this topic may help elucidate the pathophysiology of PE.

### Limitations

This study has several limitations. First, this was a single-center, cross-sectional study with a relatively small sample size. Second, the study population had specific inclusion and exclusion criteria that may have led to selection bias and limited generalizability. Third, the absence of detailed data regarding key lifestyle and psychological confounding factors (e.g., chronic stress, anxiety, and substance use) means that the observed correlation may be subject to residual confounding. Fourth, due to the observational nature of our study design, we cannot definitively establish causal inference from the observed relationships. Large-scale, prospective studies are needed to better reveal the causal relationship.

## 5. Conclusions

In this study, the frequency of the NDBP pattern was significantly higher in the PE group among normotensive individuals. This study is the first to demonstrate a significant relationship between PE and NDBP, which is closely linked to the ANS. ANS dysfunction may contribute to the etiopathogenesis of both PE and the NBDP pattern via similar mechanisms. Overall, a multidisciplinary approach can improve understanding of the link between PE and the cardiovascular system and support the development of more comprehensive diagnostic and treatment strategies.

## Figures and Tables

**Table 1:** Comparison of the demographic and clinical characteristics between the PE group and the controls.

	Controls (*n* = 80)	PE Group (*n* = 80)	*p*-Value
Age (years)	33.19 ± 5.85	33.36 ± 5.84	0.850
Body Mass Index (kg/m^2^)	26.51 ± 3.23	26.99 ± 3.34	0.345
Smoking *n* (%)	28 (35)	31 (38.7)	0.743
Fasting Glucose (mg/dL)	96.45 ± 5.52	96.73 ± 5.87	0.617
Serum Creatinine, (mg/dL)	0.89 ± 0.18	0.88 ± 0.14	0.909
Uric Acid (mg/dL)	5.09 ± 1.36	5.14 ± 1.24	0.738
ALT	22.53 ± 10.94	22.15 ± 8.56	0.302
AST	20.63 ± 6.59	20.61 ± 8.42	0.469
Total Cholesterol (mg/dL)	192.86 ± 28.13	190.05 ± 30.24	0.543
Triglyceride (mg/dL)	156.79 ± 80.15	155.03 ± 77.84	0.888
HDL-C (mg/dL)	47.11 ± 10.76	46.41 ± 10.57	0.679
LDL-C (mg/dL)	115.90 ± 27.01	114.71 ± 26.91	0.781
White Blood Cell (10^3^/µL)	7.74 ± 2.18	8.22 ± 1.78	0.121
Hgb (g/dL)	14.84 ± 1.59	14.71 ± 1.82	0.616
Platelet (10^3^/µL)	255.09 ± 71.64	258.83 ± 60.96	0.722
Systolic BP (24 h average) (mmHg)	126.41 ± 4.23	126.31 ± 5.07	0.930
Diastolic BP (24 h average) (mmHg)	81.80 ± 2.78	81.21 ± 3.63	0.607
Systolic BP (daytime) (mmHg)	130.29 ± 4.79	129.59 ± 5.45	0.487
Diastolic BP (daytime) (mmHg)	83.76 ± 2.91	83.09 ± 3.72	0.440
Systolic BP (sleep) (mmHg)	116.15 ± 5.61	118.08 ± 7.31	0.064
Diastolic BP (sleep) (mmHg)	74.95 ± 3.03	76.02 ± 4.01	0.058
Non-dipper BP pattern *n* (%)	22 (28)	39 (48)	0.009
PEDT Score	3.50 ± 2.23	14.95 ± 2.81	<0.001

Data are presented as mean ± SD, absolute and relative frequencies. A *p*-value of <0.05 was considered statistically significant. PE: premature ejaculation, ALT: alanine transaminase, AST: aspartate aminotransferase, HDL-C: high-density lipoprotein cholesterol, LDL-C: low-density lipoprotein cholesterol, Hgb: hemoglobin, BP: blood pressure, PEDT: premature ejaculation diagnostic tool.

**Table 2:** Multivariate logistic regression analysis results.

	Beta	Standard Error	*p*-Value	Odds Ratio	95% CI for Odds Ratio	
					Lower	Upper
NDBP	0.919	0.336	0.006	0.399	0.207	0.770
WBC	0.115	0.083	0.163	1.122	0.954	1.320

Multivariate logistic regression analysis by using the premature ejaculation as the dependent variable, the binary for NDBP and original input for WBC as independent variables. CI: confidence interval, NDBP: non-dipper blood pressure, WBC: white blood cell.

## Data Availability

The data of this study can be requested from the corresponding author when certain acceptance reasons are presented.

## Abbreviations

The following abbreviations are used in this manuscript:

PE	Premature ejaculation
ANS	Autonomic nervous system
BP	Blood pressure
DBP	Dipper blood pressure
NDBP	Non-dipper blood pressure
LST	Lumbar spinothalamic
SNS	Sympathetic nervous system
PNS	Parasympathetic nervous system
CVD	Cardiovascular disease
ABPM	Ambulatory blood pressure measurement
PEDT	Premature ejaculation diagnostic tool
IELT	Intravaginal ejaculatory latency time
BMI	Body mass index
